# Supplementary material for: Evaluation of Xpert® MTB/XDR test for susceptibility testing of Mycobacterium tuberculosis to first and second-line drugs in Uganda
Source: medRxiv. 2023 Apr 5:2023.04.03.23288099. Preprint. [Version 1] doi: 10.1101/2023.04.03.23288099 (PMC10104194; doi:10.1101/2023.04.03.23288099)
Supplement: 1 [file NIHPP2023.04.03.23288099v1-supplement-1.pdf]

medRxiv preprint doi: https://doi.org/10.1101/2023.04.03.23288099; this version posted April 5, 2023. The copyright holder for this preprint (which was not certified by peer review) is the author/funder, who has granted medRxiv a license to display the preprint in perpetuity.
It is made available under a CC-BY 4.0 International license .

Supporting information

S1File: Dataset for the validation of Xpert MTB/XDR test in Uganda
